# Mammarenaviruses of Rodents, South Africa and Zimbabwe

**DOI:** 10.3201/eid2712.211088

**Published:** 2021-12

**Authors:** Antoinette A. Grobbelaar, Jocelyn Jardine, Felicity J. Burt, Alasdair J. Shepherd, Susan P. Shepherd, Patricia A. Leman, Alan Kemp, Lawrence E.O. Braack, Jacqueline Weyer, Janusz T. Paweska, Robert Swanepoel

**Affiliations:** National Institute for Communicable Diseases, Johannesburg, South Africa (A.A. Grobbelaar, J. Jardine, F.J. Burt, A.J. Shepherd, S.P. Shepherd, P.A. Leman, A. Kemp, J. Weyer, J.T. Paweska, R. Swanepoel);; South African National Parks Board, Skukuza, South Africa (L.E.O. Braack).

**Keywords:** mammarenavirus, South Africa, Zimbabwe, *Mastomys natalensis*, Mopeia virus, viruses, rodents, Natal multimammate mouse

## Abstract

We conducted a survey for group-specific indirect immunofluorescence antibody to mammarenaviruses by using Lassa fever and Mopeia virus antigens on serum specimens of 5,363 rodents of 33 species collected in South Africa and Zimbabwe during 1964–1994. Rodents were collected for unrelated purposes or for this study and stored at −70°C. We found antibody to be widely distributed in the 2 countries; antibody was detected in serum specimens of 1.2%–31.8% of 14 species of myomorph rodents, whereas 19 mammarenavirus isolates were obtained from serum specimens and viscera of 4 seropositive species. Phylogenetic analysis on the basis of partial nucleoprotein sequences indicates that 14 isolates from *Mastomys natalensis*, the Natal multimammate mouse, were Mopeia virus, whereas Merino Walk virus was characterized as a novel virus in a separate study. The remaining 4 isolates from 3 rodent species potentially constitute novel viruses pending full characterization.

In response to the emergence of Lassa fever, Marburg virus, and Ebola virus in Africa, a Biosafety Level 4 laboratory was constructed at the National Institute for Communicable Diseases (NICD) in Johannesburg, South Africa, and became operational in 1980 (*1*). To establish which known viral hemorrhagic fevers in Africa occurred in South Africa and neighboring countries, antibody surveys were conducted on selected human, livestock, and wild animal populations. Findings for Crimean-Congo hemorrhagic fever were reported (*2*,*3*), but subsequent engagement of the laboratory in the investigation of a series of hemorrhagic fever outbreaks in Africa led to the suspension of survey publication. We present the results of a survey of 5,363 rodents for evidence of infection with mammarenaviruses and details of the isolation of mammarenaviruses from seropositive species. This project was undertaken with approval of the Ethics Committee of the National Institute for Virology, subsequently incorporated into NICD. 

## Materials and Methods

### Viruses, Antigens, Antiserum, and Antibody Tests

We prepared antigen slides to screen for group-specific antibody activity to mammarenaviruses by indirect immunofluorescence (IF) with Mopeia virus (MOPV) AN20410 and Lassa virus (LASV) Josiah (Table 1) grown in Vero 76 cells as described previously (*4*). The tests were performed with commercially available antimouse immunoglobulin fluorescein conjugate or recombinant protein A/G conjugate (both ThermoFisher Scientific, https://www.thermofisher.com) for nonmyomorph species. Polyclonal control antiserum was prepared by intraperitoneal inoculation of mice with live virus and exsanguination 6 weeks later. We screened serum specimens at dilutions of 1:8 and 1:16, titrated positive samples to endpoint, and confirmed the result by ELISA with MOPV antigen.

**Table 1:** Origins of viruses and nucleotide sequences used in serologic and phylogenetic studies of mammarenaviruses of rodents, South Africa and Zimbabwe*

Virus isolate	Source	Location	Obtained from	Latitude†	Longitude†	GenBank accession no.‡
Mopeia SPU84/491/40	*Mastomys natalensis*	Nwaswitshaka, South Africa	This study	−24.875	31.625	KF926396
Mopeia SPU84/491/38	*M*. *natalensis*	Nwaswitshaka, South Africa	Isolated in this study	−24.875	31.625	Not on tree§
Mopeia SPU84/491/45	*M*. *natalensis*	Nwaswitshaka, South Africa	Isolated in this study	−24.875	31.625	Not on tree§
Mopeia SPU84/491/74	*M*. *natalensis*	Nwaswitshaka, South Africa	Isolated in this study	−24.875	31.625	Not on tree§
Mopeia SPU84/491/75	*M*. *natalensis*	Nwaswitshaka, South Africa	Isolated in this study	−24.875	31.625	Not on tree§
Mopeia SPU84/491/50	*M*. *natalensis*	Nwaswitshaka, South Africa	Isolated in this study	−24.875	31.625	KF926397
Mopeia SPB801480	*M*. *natalensis*	Kwekwe, Zimbabwe	CDC			KF926400
Mopeia SPU84/491/69	*M*. *natalensis*	Nwaswitshaka, South Africa	Isolated in this study	−24.875	31.625	KF926399
Mopeia SPU84/491/73	*M*. *natalensis*	Nwaswitshaka, South Africa	Isolated in this study	−24.875	31.625	KF926401
Mopeia SPU84/491/52	*M*. *natalensis*	Nwaswitshaka, South Africa	Isolated in this study	−24.875	31.625	KF926398
Mopeia SPU89/462/61	*M*. *natalensis*	Mahudzi, South Africa	Isolated in this study	−23.875	31.625	KF926405
Mopeia SPU94/088/235	*M*. *natalensis*	Mazithi, South Africa	Isolated in this study	−24.375	31.875	KF926406
Mopeia SPU94/089/38	*M*. *natalensis*	Nhlanganzwani, South Africa	Isolated in this study	−25.125	31.875	KF926404
Mopeia SPU94/089/33	*M*. *natalensis*	Nhlanganzwani, South Africa	Isolated in this study	−25.125	31.875	KF926403
Mopeia SPU82/030/1	*M*. *natalensis*	Flesk, Zimbabwe	Isolated in this study	−20.125	30.875	KF926407
Mopeia AN20410	*M*. *natalensis*	Mopeia, Mozambique	Reisolated in this study	−18.037	35.733	KF926391
Mopeia AN21366	*M*. *natalensis*	Mopeia, Mozambique	Reisolated in this study	−17.808	35.733	KF926395
Mopeia AN20602	*M*. *natalensis*	Mopeia, Mozambique	Reisolated in this study	−18.037	35.733	KF926392
Mopeia AN20615	*M*. *natalensis*	Mopeia, Mozambique	Reisolated in this study	−18.037	35.733	KF926393
Morogoro 3017/2004	*M*. *natalensis*	Magadu, Tanzania	GenBank, sequence only	NA	NA	EU914103
Mopeia SPB801478	*M*. *natalensis*	Chiredzi, Zimbabwe	CDC	NA	NA	KF926402
Mopeia AN20616	*M*. *natalensis*	Mopeia, Mozambique	Reisolated in this study	−18.037	35.733	KF926394
Gairo TZ-27421	*M*. *natalensis*	Tanzania	GenBank, sequence only	NA	NA	KJ855308
Dhati Welel LAV2586	*M*. *natalensis*	Ethiopia	GenBank, sequence only	NA	NA	MT078838
Mobala ACAR 3080	*Praomys jacksoni*	Central African Republic	GenBank, sequence only	NA	NA	AY342390
Luna NMW-1	*Aethomys chrysophilus*	Zambia	GenBank, sequence only	NA	NA	AB586646
Gbagroube CIV6008	*Mus setulosus*	Cote d'Ivoire	GenBank, sequence only	NA	NA	GU830848
Jirandogo JIR76	*Mus baoulei*	Ghana	GenBank, sequence only	NA	NA	JX845169
Lassa 391	Human	Nigeria	GenBank, sequence only	NA	NA	X52400
Lassa Josiah	Human	Sierra Leone	CDC	NA	NA	J04324
Lassa 331	Human	Sierra Leone	CAMR	NA	NA	KF926390
Menekre CIV1227	*Hylomyscus* spp.	Cote d’Ivoire	GenBank, sequence only	NA	NA	GU830862
Solwezi 13ZR68	*Grammomys* spp.	Zambia	GenBank, sequence only	NA	NA	AB972428
Ippy DakAnB188d	*Arvicanthus* spp.	Central African Republic	YARU	NA	NA	DQ328877
Mariental N27 MRMi. N9	*Micaelamys namaquensis*	Namibia	GenBank, sequence only	NA	NA	KM272987
Bobomene SPU84/491/106	*A*. *chrysophilus*	Bobomene, South Africa	Isolated in this study	−22.625	31.125	KF926408
Merino Walk SPU85/353	*Otomys unisulcatus*	Merino Walk, South Africa	Isolated in this study	−31.125	27.625	KF926409
Okahandja N73 OkhMi.n4	*Micaelamys namaquensis*	Namibia	GenBank, sequence only	NA	NA	KM272988
Witsand SPU86/485	*M*. *namaquensis*	Witsand, South Africa	Isolated in this study	−28.625	22.375	KF926412
Bitu ANG0070	*M*. *namaquensis*	Tundavala, Angola	GenBank, sequence only	NA	NA	MZ065539
Omdraaivlei SPU86/415/4	*O*. *unisulcatus*	Omdraaivlei, South Africa	Isolated in this study	−30.125	23.125	KF926410
Omdraaivlei SPU86/415/2	*O*. *unisulcatus*	Omdraaivlei, South Africa	Isolated in this study	−30.125	23.125	KF926411
Souris 05775–5302–5304	*Praomys sp.*	Cameroon	GenBank, sequence only	NA	NA	KP050227
Natorduori NAT23	*Mus mattheyi*	Ghana	GenBank, sequence only	NA	NA	JX845170
Lunk NKS-1	*Mus minutoides*	Zambia	GenBank, sequence only	NA	NA	NC018710
Dandenong 0710–2678	Human	Australia	GenBank, sequence only	NA	NA	EU136038
LCM WE	Human	New York, USA	GenBank, sequence only	NA	NA	M22017
LCM Armstrong 53	Human	Missouri, USA	GenBank, sequence only	NA	NA	M20869
Lujo SPU08/308	Human	Zambia	NICD	NA	NA	FJ952384
Tacaribe 11573	*Artibeus lituratus* (bat)	Trinidad	GenBank, sequence only	NA	NA	M20304
Pichinde 3739	*Oryzomys albigularis*	Colombia	GenBank, sequence only	NA	NA	K02735
Oliveros 3229–1	*Bolomys obscurus*	Argentina	GenBank, sequence only	NA	NA	U34248

*CAMR, Center for Applied Microbiology and Research; CDC, US Centers for Disease Control and Prevention; NA, not applicable; NICD, National Institute for Communicable Diseases; USAMRIID, United States Army Research Institute of Infectious Diseases; YARU, Yale Arbovirus Research Unit. †Geographic coordinates are shown for viruses isolated or reisolated from field samples in this study. ‡GenBank accession numbers are shown for partial nucleotide sequences used for phylogenetic analysis in Figure 5. §Same partial nucleotide sequences as isolate SPU84/491/40.

Cell lysate antigen for the ELISA was prepared and assays conducted as described previously for Ebola virus (*5*), by using antimouse horseradish peroxidase–conjugated IgG (SeraCare Life Sciences, Inc., https://www.seracare.com). In the absence of control data, we recorded reactions as positive where the net optical density of test serum specimens at 1:100 was >2.5 times the mean optical density of a panel of serum specimens from specific pathogen-free laboratory mice. Monoclonal antibodies to LASV and MOPV were obtained from the US Centers for Disease Control and Prevention (CDC; Atlanta, GA, USA) or prepared at NICD as described elsewhere for Crimean-Congo hemorrhagic fever virus (Table 4) (*6*).

**Table 4:** Reactivity of selected mammarenavirus isolate-infected cell antigens in indirect immunofluorescence tests with monoclonal antibodies to Lassa fever and Mopeia viruses, South Africa and Zimbabwe*

Virus antigen	Monoclonal antibody titer†						
	CDC 5254–6 Lassa N	CDC 5293–4 Lassa N	CDC 5273–8 Lassa N	CDC 5285–6 Lassa G	CDC 5329–1 Mopeia N	NICD 4E9 Mopeia N	NICD 3G9 Mopeia N
LCM Armstrong	>12,800	–	–	1,600	–	–	–
Lassa Josiah	>12,800	>12,800	>12,800	3,200	–	>12,800	–
Ippy DakAnB 188d	>12,800	–	–	3,200	3,200	>12,800	–
Mobala A11/3076	>12,800	–	–	6,400	–	>12,800	–
Mopeia AN 20410	>12,800	>12,800	–	400	>12,800	>12,800	>12,800
Mopeia SPU82/30/1	>12,800	>12,800	400	800	>12,800	>12,800	>12,800
Mopeia SPU84/491/73	>12,800	>12,800	400	200	>12,800	>12,800	>12,800
Bobomene SPU84/491/106	>12,800	>12,800	–	1,600	>12,800	>12,800	>12,800
Witsand SPU86/485	>12,800	>12,800	>12,800	–	>12,800	>12,800	–
Omdraaivlei SPU86/415/2	>12,800	>12,800	>12,800	–	>12,800	>12,800	–
Merino Walk SPU85/353	>12,800	–	–	–	>12,800	–	–

*G, antiglycoprotein; N, antinucleoprotein; –, none. †Antibody titers shown as reciprocals of serum dilution.

### Rodent Samples and Virus Isolation Studies

Most samples were opportunistically derived from material collected for unrelated surveys and stored at NICD. The initial 213 samples were collected at NICD during 1964–1981 for arbovirus surveys, 3,542 samples were collected and submitted by the Department of Health of South Africa during 1971–1988 for plague surveillance in the central part of the country, 831 rodents (with an emphasis on *Mastomys natalensis* mice) were collected in northeastern South Africa during 1984–1994 specifically for the investigation of mammarenaviruses, 764 rodent samples collected in Zimbabwe in 1974 were remnants of a study on Rift Valley fever virus (*7*), and 13 samples were collected in 1982 on a farm in south-central Zimbabwe where there had been a suspected but unconfirmed case of viral hemorrhagic fever in a patient admitted to a hospital in South Africa. Live-trapped rodents were euthanized and exsanguinated; serum samples and visceral organ (lung, heart, liver, spleen, and kidney) samples were conveyed to NICD with ice packs and stored at −70°C. Coordinates of sample collection sites were recorded as quarter-degree grid cells.

We confirmed identities of rodent species yielding virus isolates by determining partial cytochrome b gene sequences for 8 selected samples (*8*). Skull and skin preparations of rodents from plague surveillance were deposited in the Ditsong National Museum of Natural History (Pretoria, South Africa), and selected materials from other surveys were preserved at NICD.

We attempted isolation of mammarenaviruses for rodent species at locations where antibody was found. We inoculated serum and 10% clarified suspensions of pooled viscera onto Vero 76 monolayer cultures in replicate Lab-Tek 8-chamber slides (ThermoFisher Scientific) and examined after incubation for 7–10 days at 37°C by IF with pooled mouse antiserum to MOPV and LASV. We passed samples 3 times before recording them as negative. The 5 original isolates of MOPV from *M. natalensis* rodents from Mozambique were taken to CDC in 1977 (*9*); we used duplicate organ samples stored at NICD to reisolate the viruses. We screened antigen cell spots prepared from cultures infected with selected known mammarenaviruses plus isolates from this study by IF against mammarenavirus monoclonal antibodies at doubling dilutions from 1:100. We tested all isolates for intracerebral pathogenicity for 1-day-old mice by inoculation of 2 litters (8 infant mice/litter) for each virus.

### Molecular Characterization and Phylogenetic Analysis of Mammarenavirus Isolates

We performed phylogenetic analysis on 48 isolates by using an ≈912-nt (299–304-aa) fragment of the nucleocapsid protein (NP) gene, consisting of 15 isolates from this study, 5 MOPV isolates from Mozambique (*9*) that were reisolated during this study, 3 mammarenaviruses received from other laboratories—namely, LASV 331 and MOPV isolates SPB801478 and SPB801480 from Zimbabwe (*10*)—and 25 viruses for which nucleotide sequences were retrieved from GenBank, including 3 New World arenaviruses as outgroup taxa (Table 1). We omitted from analysis 4 isolates from this study with identical sequences to Mopeia virus isolate SPU84/491/40 (Table 1). We excluded potential related viruses for which inadequate information was available, such as Kodoko virus from Guinea (*11*) and Lemniscomys virus from Tanzania (*12*).

We extracted total RNA from cultures by using the High Pure RNA isolation kit (Roche Diagnostics, https://www.roche.com) and performed reverse transcription PCR with primers 19C (5′-CGCACAGTGGATCCTAGGC-3′) (*13*) and OWA_2_ (5′-TTCTTCATAAGGGTTCCTTTCACC-3′) (J.C.S. Clegg, Centre for Applied Microbiology and Research, pers. comm., 1991) by using the Titan One Tube reverse transcription PCR kit (Roche) to amplify an ≈1,000-bp fragment of the NP gene. The 19C primer is complementary to a conserved sequence at the 3′ terminus of the S RNA segment and the OWA_2_ primer corresponds to nucleotide positions 2402–2424 relative to LASV 391 (GenBank accession no. X52400). We designed a degenerate reverse primer, Arena A (5′-ATRTARGGCCAWCCSTCTCC-3′), corresponding to nucleotide positions 2357–2376 relative to LASV 391 and 2401–2420 relative to MOPV AN20410 (GenBank accession no. NC006575), to amplify the region of interest for isolates SPU94/88/235 and SPU86/485. Cycling conditions were 50°C for 30 min, 94°C for 2 min, 30 cycles of 94°C for 30 s, 47°C for 30 s, and 68°C for 90 s, plus extension at 68°C for 7 min. We purified PCR products with the Wizard SV Gel and PCR clean-up kit (Promega Corporation, https://www.promega.com), sequenced with the BigDye Terminator v3.1 Cycle Sequencing Kit (ThermoFisher Scientific), purified on Centrisep columns (Princeton Separations Inc., https://www.prinsep.com), and ran testing on an ABI Prism 377 DNA Sequencing Unit. We aligned nucleotide and predicted amino acid sequences using ClustalW (http://www.clustal.org/clustal2) incorporated in MEGA7 (https://www.megasoftware.net), performed phylogenetic analysis by the neighbor-joining method with 1,000 bootstrap iterations, and calculated sequence diversities (p-distances) (*14*). 

## Results

**Figure 1:**
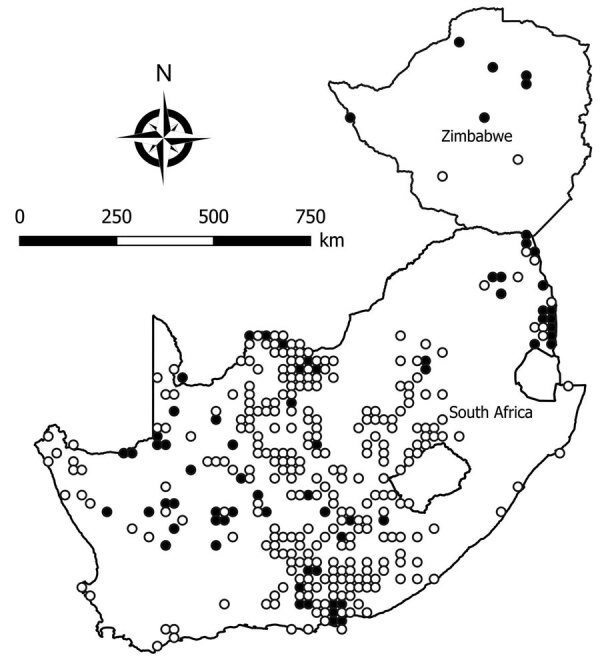
Locations where samples from 5,363 rodents of 33 species were collected and tested for evidence of infection with mammarenaviruses, South Africa and Zimbabwe. White circles indicate sites where no evidence of infection was found; black circles indicate sites where antibody to mammarenaviruses was detected by indirect immunofluorescence.

**Figure 2:**
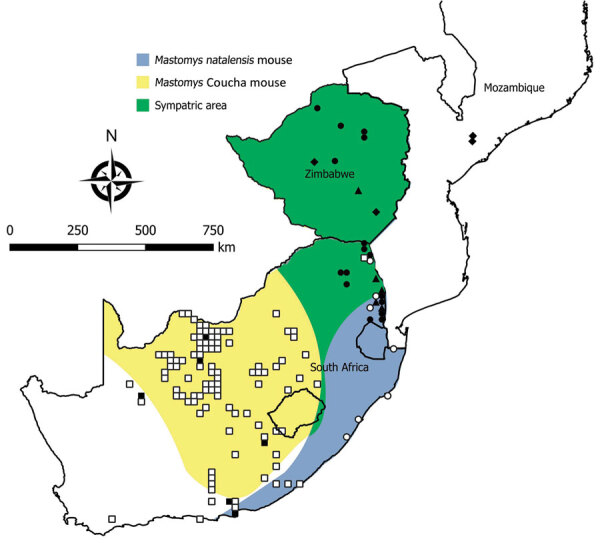
Locations where samples were collected from *Mastomys* spp. rodents, South Africa and Zimbabwe. White squares indicate sites where no antibody to mammarenaviruses was found in *M. coucha* mouse serum specimens; black squares,where antibody was detected in *M. coucha* mouse serum specimens; white circles, where no antibody to mammarenaviruses was found in *M. natalensis* mouse serum specimens; black circles, where antibody was detected in *M. natalensis* mouse serum specimens; black triangles, where Mopeia virus was isolated from *M. natalensis* mouse samples during this study; black diamonds, where Mopeia virus was isolated from *M. natalensis* mouse samples during previous studies, including the original isolations in Mozambique (*9*,*10*). Shading indicates distribution ranges for *M. coucha* and *M. natalensis* mice. Adapted from Chimimba and Bennett (*15*).

**Figure 3:**
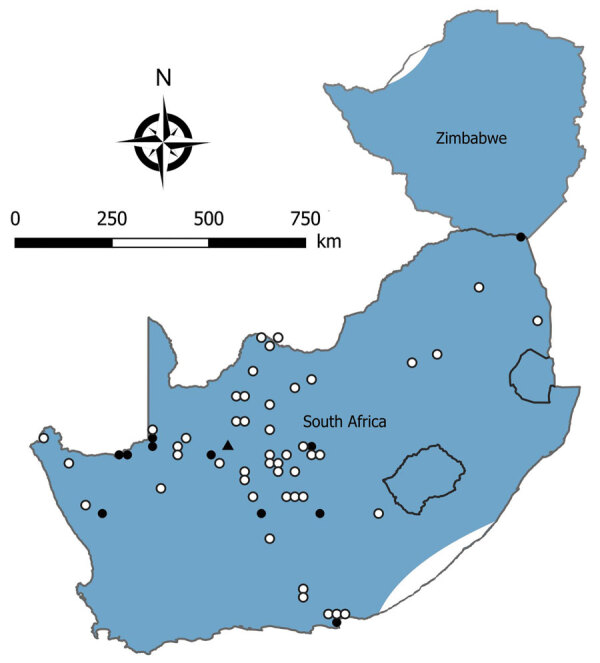
Locations where samples were collected from *Micaelamys namaquensis* rodents, South Africa and Zimbabwe. White circles indicate sites where no antibody to mammarenaviruses was found in *M. namaquensis* rat serum specimens; black circles, where antibody was detected in *M. namaquensis* rat serum specimens; black triangle, where a mammarenavirus isolate was obtained from an *M. namaquensis* rat sample. Shading indicates distribution range of *M. namaquensis* rats. Adapted from Chimimba and Bennett (*15*).

**Figure 4:**
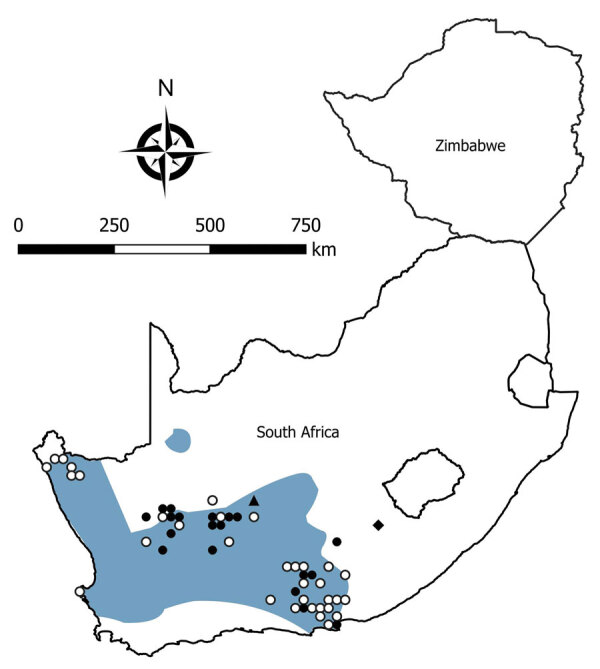
Locations where samples were collected from *Otomys unisulcatus* rodents, South Africa and Zimbabwe. White circles indicate sites where no antibody to mammarenaviruses was found in *O. unisulcatus* rat serum specimens; black circles, where antibody was detected in *O. unisulcatus* rat serum specimens; black triangle, where Omdraaivlei mammarenavirus isolates were obtained from *O. unisulcatus* rat samples; black diamond, where Merino Walk virus was isolated from *O. unisulcatus* rat. Shading indicates distribution range of *O. unisulcatus* rats. Adapted from Chimimba and Bennett (*15*).

We tested a total of 5,363 rodents of 33 species from collection sites throughout South Africa and Zimbabwe for antibody to mammarenaviruses (Table 3; Figures 1,2,3,4). Antibody was found to be widely distributed in the 2 countries (Figures 1,2,3,4) and was detected in serum samples of 1.2%–31.8% of 14 species of myomorph rodents; 19 mammarenavirus isolates were obtained from serum and viscera of 4 seropositive species (Table 3).

**Table 3:** Summary of rodent samples, serologic test results, and virus isolation studies in study of mammarenaviruses, South Africa and Zimbabwe*

Rodent species scientific name	Rodent species common name	Mammarenavirus IF antibody tests				Mammarenavirus isolation attempts			
		Tested	No. (%) positive	Titer range (GMT)		Serum samples	Organs	Total	Isolations
*Aethomys chrysophilus*	Red veld rat	135	10 (7.4)	8–4,096 (207.8)		23	75	77	1
*A. ineptus*	Tete veld rat	103	0			0	0	0	0
*Dasymys incomtus*	Water rat	2	0			0	1	1	0
*Dendromus melanotis*	Grey climbing mouse	1	0			0	1	1	0
*Desmodillus auricularis*	Short-tailed gerbil	69	0			0	3	3	0
*Gerbilliscus afra*	Cape gerbil	26	0			6	10	13	0
*G. brantsii*	Highveld gerbil	529	4 (0.8)	8–256 (26.9)		0	14	14	0
*G. leucogaster*	Bushveld gerbil	378	14 (3.7)	8–1,024 (57.9)		8	42	42	0
*G. paeba*	Hairy-footed gerbil	8	0			2	1	2	0
*Lemniscomys rosalia*	Single-striped mouse	21	0			2	8	8	0
*Mastomys coucha*	Cape multimammate mouse	664	11 (1.7)	8–512 (56.4)		14	63	73	0
*M. natalensis*	Natal multimammate mouse	1165	370 (31.8)	8–16,384 (219.5)		151	307	380	14
*Micaelamys namaquensis*	Namaqua rock mouse	273	20 (7.3)	8–2,048 (132.4)		44	85	101	1
*Mus minutoides*	Pygmy mouse	16	0			0	28	28	0
*M. musculus*	House mouse	25	0			0	23	23	0
*Mystromys albicaudatus*	White-tailed mouse	11	0			0	1	1	0
*Otomys angoniensis*	Angoni vlei rat	30	1 (3.3)	16		0	3	3	0
*O. irroratus*	Vlei rat	266	67 (25.2)	8–8,192 (127.9)		315	59	346	0†
*O. unisulcatus*	Bush vlei rat	178	28(21.3)	8–2,048 (99.1)		17	19	28	3
*Parotomys brantsii*	Brant’s whistling rat	10	1 (10.0)	256		0	0	0	0
*P. littledalei*	Littledale’s whistling rat	3	0	0		0	0	0	0
*Rattus norvegicus*	Brown rat	125	6 (4.8)	8–8,192 (644.6)		8	47	48	0
*R. rattus*	House rat	211	1 (0.5)	512		6	38	38	0
*Rhabdomys pumilio s.l.*	Four-striped mouse	933	11 (1.2)	8–64 (19.3)		73	59	118	0
*Saccostomus campestris*	Pouched mouse	82	6 (7.3)	8–1,024 (25.4)		11	24	28	0
*Steatomys pratensis*	Fat mouse	3	0			0	2	2	0
*Thallomys paedulcus*	Tree mouse	31	0			11	27	27	0
*Zelotomys woosnami*	Woosnam’s desert rat	1	0			0	0	0	0
*Cryptomys hottentotus*	Common mole rat	6	0						
*Graphiurus murinus*	Woodland doormouse	8	0						
*Paraxerus cepapi*	Tree squirrel	6	0						
*Xerus inauris*	Ground squirrel	36	0						
*Pedetes capensis*	Springhare	8	0						
Totals		5,363	560			691	940	1,405	19

*IF, immunofluorescence. †Organ pool from 1 *O. irroratus* rat was positive in mammarenavirus IF on first pass in culture but the potential isolate was lost on subculture.

Identities of the 4 myomorph species that yielded mammarenavirus isolates—*M. natalensis* mice and *Aethomys chrysophilus*, *Micaelamys namaquensis*, and *Otomys unisulcatus* rats—were confirmed from partial cytochrome b gene sequences (*8*) (GenBank accession nos. MK531528–35). However, the genus *Micaelamys* has subsequently proved to be polyphyletic and due for revision (*16*), whereas there is debate about inclusion of *O. unisulcatus* in the genus *Myotomys* (*17*). Furthermore, *O. unisulcatus* tissue remained available only for the Omdraaivlei isolates and not for the Merino Walk isolate. Most of the other myomorph rodents were identified from morphologic features and distribution patterns (*18*), but new species and subspecies with partially overlapping distributions have since been recognized in the genus *Rhabdomys* (*19*–*21*). No organs remained available, and serum specimens failed to yield DNA for phylogenetic studies; thus, the samples are recorded as *R. pumilio* sensu lato (Table 3).

No mammarenavirus antibody or virus was found in 14 of the myomorph rodent species (Table 3), and although these rodents were relatively poorly represented in the collection, they tend to be rare species or occur in specialized habitats, such as deserts. A further 9 species of rodents—*M. coucha*, *O. angoniensis*, *Parotomys brantsii*, *Rattus norvegicus*, *R. rattus*, *R. pumilio* s.l., *Saccostomus campestris*, *Gerbilliscus brantsii*, and *G. leucogaster*—had low prevalence (0.5%–7.3%) of IF antibody to mammarenaviruses; no clear tendency to cluster was noted, except that the reactions were detected in locations where antibody was prevalent in other species. Although IF titers were generally low in these species (geometric mean titers [GMT] 19.3–57.9), the few >16 tended to be supported by ELISA reactions, but samples cultured yielded no virus. Anomalous high IF titers of 8,192 supported by ELISA reactions were recorded in 2 serum samples from *R. norvegicus* rats collected from a location where antibody prevalence of 29.1% was recorded in serum specimens from *O. irroratus* rats, but no isolates were obtained.

The remaining 5 species of myomorph rodents—*A. chrysophilus*, *M. namaquensis*, *M. natalensis*, *O. unisulcatus*, and *O. irroratus*—had mammarenavirus IF antibody prevalence of 4.2%–31.8%; positive reactions tended to cluster and reached 30%–50% prevalence at some trapping sites. The IF titers ranged from 8–16,384 (GMT 99.1–219.5), and titers >16 were supported by positive ELISA reactions. A total of 19 mammarenavirus isolates were obtained from 4 of these species (Table 3), but a single sample of *O. irroratus* rat produced an IF reaction on first pass in cell cultures that was lost during subculture and could not be repeated in further attempts to isolate virus. In addition, attempts to reisolate MOPV from 5 sets of *M. natalensis* organs from Mozambique (*9*) held in storage at NICD were successful (Table 1). All isolates were pathogenic for day-old mice inoculated intracerebrally.

**Figure 5:**
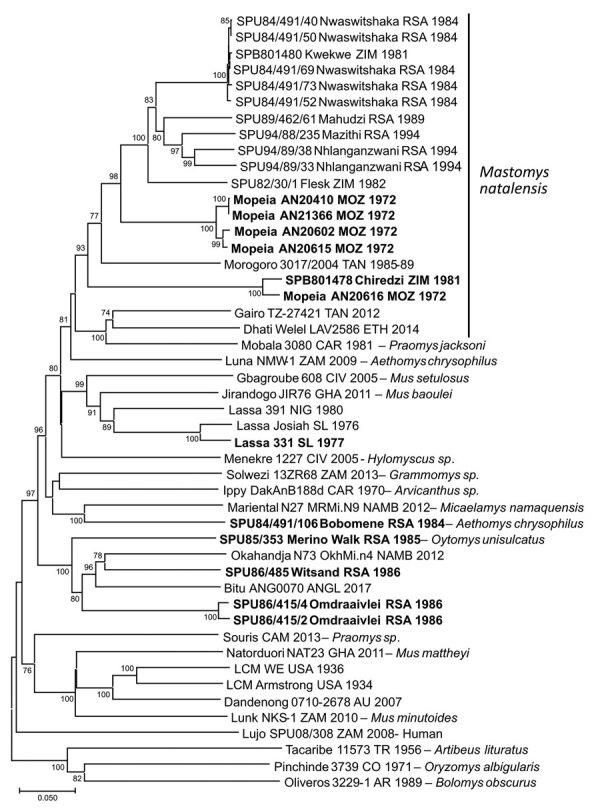
Phylogenetic relationships of 48 arenavirus isolates based on neighbor-joining analysis applying a Jukes-Cantor model of partial nucleoprotein sequences (≈912 nt), together with known host relationships and collection dates. Values at nodes indicate the level (%) of bootstrap support from 1,000 replicates. Scale bar indicates base substitutions per site. Bold indicates sequences determined in this study. ANGL, Angola; AR, Argentina; AU, Australia; CAM, Cameroon; CAR, Central Africa Republic; CO, Colombia; CIV, Côte d'Ivoire; ETH, Ethiopia; GHA, Ghana; MOZ, Mozambique; NAMB, Namibia; NIG, Nigeria; RSA, Republic of South Africa; SL, Sierra Leone; TAN, Tanzania; TR, Trinidad; USA, United States; ZAM, Zambia; ZIM, Zimbabwe.

**Figure 6:**
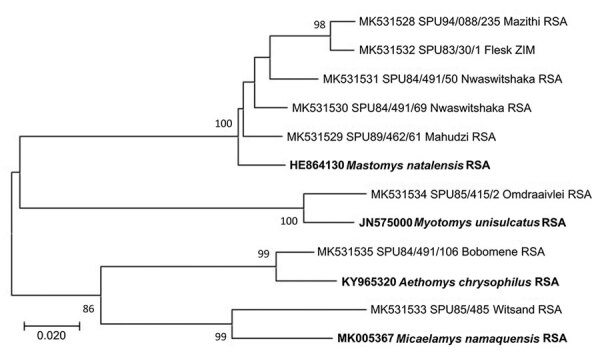
Phylogenetic relationships of 8 rodents from which mammarenavirus isolates were obtained in this study (bold) and reference taxonomic voucher sequences from GenBank. Tree was constructed based on neighbor-joining analysis of a 136-bp cytochrome b barcode sequence. Values at nodes indicate the level of bootstrap support from 1,000 replicates. Scale bar indicates base substitutions per site. GenBank accession numbers, rodent reference number, and country of collection are indicated. RSA, Republic of South Africa; ZIM, Zimbabwe.

A total of 6 isolates from this study plus 1 reisolated MOVP from Mozambique demonstrated 4 patterns of reactivity in IF screening tests with monoclonal antibodies and selected mammarenavirus isolates (Table 4). Deduced NP amino acid distances between selected isolates and closest relatives were calculated (Table 2). We determined the phylogenetic relationships of 48 mammarenavirus isolates, including 15/19 isolates from this study and the 5 reisolated MOPV isolates from Mozambique (Table 1), on the basis of neighbor-joining analysis of partial NP sequences (≈912 nt), together with host relationships (Figure 5). The *M. natalensis* isolates from Mozambique and from this study grouped with 2 earlier isolates from Zimbabwe as Mopeia virus, whereas 5 isolates from this study fell into 4 groups; isolate Bobomene from South Africa grouped with more recent isolates Mariental from Namibia and isolate Witsand from South Africa grouped with Okahandja from Namibia and with isolate Bitu from Angola (Figure 5). We determined phylogenetic relationships on the basis of neighbor-joining analysis of a 136 bp cytochrome b barcode sequence for 8 selected rodents from which mammarenavirus isolates were obtained in this study and reference taxonomic voucher sequences from GenBank (Figure 6).

**Table 2:** Pairwise comparison of partial nucleocapsid protein amino acid sequence (299–304 aa) percentage difference between 5 selected southern Africa Old World mammarenavirus isolates from current study and closest relatives, South Africa and Zimbabwe*

Isolate name	MOPV AN20616	IPPYV DakAn B188	LASV Josiah	MOBV 3080	LCMV WE	LUJO ZAM	LUNK NKS1	LUNA NMW1	MRTV NR27	OKAV NR73	BITU ANG 0070	SPU 86/ 415/ 2	SPU 86/ 415/ 4	SPU 86/ 485	SPU 84/ 491/ 406	SPU 85/ 353
SPU 86/415/2	30	29	29.6	31.3	37.7	43.4	34.7	30	33	15.5	14.2	0	3.7	15.2	32	19.9
SPU 86/415/4	30	28.3	29	31.3	37	43.3	34.3	29.6	32	15.2	14.2	3.7	0	14.8	31.3	19.2
SPU 86/485	27.6	27.6	29.6	29.3	33	39.4	31	28.3	29.3	8.4	9.3	15.2	15	0	29.3	20.5
SPU 84/491/ 106	24	22	25	25	34	44	35	26	13	30	35.3	32	31.3	29.3	0	27.3
SPU 85/353	26.6	27.6	28.3	29.6	33	42.8	32	28.3	28.3	20.9	19.4	19.9	19.2	20.5	27.3	0

*BITU, Bitu virus; IPPYV, Ippy virus; LASV, Lassa virus; LCMV, lymphocytic choriomeningitis virus; LUJO, Lujo virus; LUNA, Luna virus; LUNK, Lunk virus; MRTV, Mariental virus; MOBV, Mobala virus; MOPV, Mopeia virus; OKAV, Okahandja virus.

## Discussion

The main impetus for this rodent survey came from the isolation of the mammarenavirus MOPV at NICD from *M. natalensis* rodents collected in a village in Mozambique during an arbovirus study in 1972; within months, the same rodent species was identified as the host of LASV in West Africa (*9*,*22*,*23*). As a consequence, work ceased on Mopeia virus at NICD and the isolates were transferred to CDC, where the relationship to LASV was confirmed (*9*). Although MOPV proved to be nonpathogenic for nonhuman primates (*24*), investigating the possible occurrence and role of mammarenaviruses as causes of human infection in South Africa was considered necessary.

Our survey detected widespread presence of antibody activity to mammarenaviruses in myomorph rodent serum specimens within the study area. Because *M. natalensis* mice have an eastern distribution in South Africa (*18*), rodents were trapped along the northeastern border and MOPV was successfully isolated. However, a mammarenavirus isolated from another rodent species, *A. chrysophilus*, within the distribution range of *M. natalensis* mice was found to be distinct from MOPV; 4 isolates obtained from 2 other rodent species further to the west also differed from MOPV (Tables 1–4; Figure 5).

Unpublished serosurveys conducted on humans in South Africa during 1984–1988 in parallel with the rodent survey included a study of 7,665 long-term (>5 years) healthcare workers from 66 secondary hospitals that receive patient referrals from district hospitals who were tested for evidence of nosocomial infection, plus a study of 2,041 long-term (>5 years) rural residents and workers in the livestock and wildlife industries who were investigated for evidence of exposure to zoonotic viruses (R. Swanepoel, unpub. data). An overall prevalence of 1.0% (93/9,704) of IF antibody to MOPV antigen was recorded at titers of 8–2,048, gmt 33.0; higher prevalences of 10%–15% occurred in a few widely separated locations near the eastern border, but no histories of disease considered indicative of mammarenavirus infection were obtained. To the west, in Free State, Northern Cape, North West, and Gauteng Provinces, no antibody to mammarenaviruses was detected in rural residents and workers in the livestock industry despite the isolation of mammarenaviruses from rodents. Aliquots of human serum samples collected during the original investigations in Mopeia village, Mozambique, in 1972 remained available at NICD, and IF antibody to MOPV antigen was detected at a prevalence of 16.1% (32/199) with titers ranging from 8 to 8,192, gmt 229.6, similar to the findings initially recorded when no disease associations were identified (R. Swanepoel, unpub. data).

In further checks on the possible occurrence of mammarenavirus-associated disease, 379 patients experiencing febrile illness in 4 district hospitals along the northeastern coast of KwaZulu-Natal Province, South Africa, were monitored for evidence of MOPV infection or seroconversion in 1985 without positive result. No antibody was detected in 100 chronic renal failure patients on dialysis in Gauteng Province, South Africa, in 1993 (R. Swanepoel, unpub. data).

Among routine diagnostic samples submitted to NICD, resting IF titers of 128 and 256 of IgG to MOPV antigen were detected in 2 patients from South Africa, but no etiologic significance could be attached to these findings. A single case of fatal LASV infection was diagnosed in a patient from Nigeria who was evacuated to a hospital in South Africa in 2007 (R. Swanepoel, unpub. data). The only other human arenavirus infections diagnosed within South Africa were in 2 patients referred successively from Zambia in 2008 who were infected with the novel Lujo virus and 3 local healthcare workers who acquired nosocomial infection from those patients (*25*). At the time of the Lujo virus outbreak, involvement of any of the mammarenaviruses isolated from rodents during the current study was ruled out; in the process, the Merino Walk isolate was characterized as a novel mammarenavirus (*26*).

The widely distributed *M. natalensis* mouse of sub-Saharan Africa consists of 6 matrilineages that fall into 2 clades, AI-III and BIV-VI, on the basis of the mitochondrial cytochrome b marker (*27*,*28*). Each lineage is associated with >1 mammarenavirus, ranging from LASV in lineage AI in West Africa to MOPV and Luna virus in lineage BVI in southern Africa (*28*,*29*). Our findings confirm the association of MOPV with *M. natalensis* mice in southern Africa, where this rodent is sympatric with *M. coucha* mice in northeastern South Africa and in Zimbabwe. However, the distribution of *M. coucha* mice extends westwards into the drier interior of South Africa; the low prevalence of MOPV antibody found in this species could represent spillover of infection from other rodents, rather than the harboring of a mammarenavirus (Table 3). Whereas *M. natalensis* mice in the mesic east are peridomestic, indigenous rodents tend to be sylvatic and less closely associated with human dwellings in the xeric west, where no evidence of infection was detected in humans.

The isolates from this study are provisionally named for their locations of origin (Table 1; Figure 5), but the isolates obtained from *M. natalensis* mice represent exemplar isolates of MOPV, and Merino Walk virus is clearly distinct. Although the apparent sharing of rodent hosts mitigates against species recognition within the mammarenaviruses (*30*), clarifying the interrelationships between the Bobomene, Witsand, and Omdraaivlei isolates and their relationship to the Mariental and Okahandja viruses from Namibia (*31*) and Bati virus from Angola (*32*) anticipates complete genomic characterization of the isolates.

The phylogenetic relationships between 8 rodents from which mammarenaviruses were isolated in this study and reference taxonomic voucher sequences from GenBank are compatible with the concept of cospeciation of arenaviruses and their rodent hosts (Figure 6), except that the interrelationships between Witsand, Okahandja, and Bitu isolates await clarification as previously noted. Moreover, the unavailability of rodent host tissue for Merino Walk virus precluded comparison with ostensibly the same host species, the *O. unisulcatus* rat, of the Omdraaivlei isolates. However, *O. unisulcatus* rats reportedly comprise a coastal lowland group that is located where the host of Merino Walk virus was collected and a central interior group that covers the area where the hosts of the Omdraaivlei isolates were obtained, although the low sequence divergences did not warrant recognition of subspecies (*33*). The observations on rodents from Zimbabwe were limited, and the single isolation of Mopeia virus obtained from *M. natalensis* mice from a farm near Masvingo was not related to the nonfatal illness of a former farm resident who was hospitalized in South Africa.

Further research on mammarenaviruses in rodents in South Africa should include attempts to isolate virus from *O. irroratus* rats and possibly *Lemniscomys rosalia* mice, which were underrepresented in this survey; the presence of Luna-related and Lunk-related viruses that were identified in Zambia in *M. natalensis* and *M. minutoides* rodents should also be investigated (*34*). Furthermore, the reservoir host and distribution range of Lujo virus in southern Africa have not been determined. A greater knowledge of the occurrence and diversity of mammarenaviruses in Africa is foundational to understanding the possible health risks associated with these viruses and preparedness for the emergence of such viruses in the future. 

## References

[1] Swanepoel R. Viral haemorrhagic fevers in South Africa: history and national strategy. S Afr J Sci. 1987;83:80–8.

[2] Swanepoel R, Shepherd AJ, Leman PA, Shepherd SP, McGillivray GM, Erasmus MJ, et al. Epidemiologic and clinical features of Crimean-Congo hemorrhagic fever in southern Africa. Am J Trop Med Hyg. 1987;36:120–32. 10.4269/ajtmh.1987.36.120 3101525

[3] Shepherd AJ, Swanepoel R, Shepherd SP, McGillivray GM, Searle LA. Antibody to Crimean-Congo hemorrhagic fever virus in wild mammals from southern Africa. Am J Trop Med Hyg. 1987;36:133–42. 10.4269/ajtmh.1987.36.133 3101526

[4] Johnson KM, Elliott LH, Heymann DL. Preparation of polyvalent viral immunofluorescent intracellular antigens and use in human serosurveys. J Clin Microbiol. 1981;14:527–9. 10.1128/jcm.14.5.527-529.1981 7031084 PMC273981

[5] Ksiazek TG, West CP, Rollin PE, Jahrling PB, Peters CJ. ELISA for the detection of antibodies to Ebola viruses. J Infect Dis. 1999;179(Suppl 1):S192–8. 10.1086/514313 9988184

[6] Blackburn NK, Besselaar TG, Shepherd AJ, Swanepoel R. Preparation and use of monoclonal antibodies for identifying Crimean-Congo hemorrhagic fever virus. Am J Trop Med Hyg. 1987;37:392–7. 10.4269/ajtmh.1987.37.392 3116871

[7] Swanepoel R, Blackburn NK, Efstratiou S, Condy JB. Studies on Rift Valley fever in some African murids (Rodentia: Muridae). J Hyg (Lond). 1978;80:183–96. 10.1017/S0022172400053535 632561 PMC2130003

[8] Galan M, Pagès M, Cosson J-F. Next-generation sequencing for rodent barcoding: species identification from fresh, degraded and environmental samples. PLoS One. 2012;7:e48374. 10.1371/journal.pone.0048374 23144869 PMC3492341

[9] Wulff H, McIntosh BM, Hamner DB, Johnson KM. Isolation of an arenavirus closely related to Lassa virus from Mastomys natalensis in south-east Africa. Bull World Health Organ. 1977;55:441–4. 304387 PMC2366678

[10] Johnson KM, Taylor P, Elliott LH, Tomori O. Recovery of a Lassa-related arenavirus in Zimbabwe. Am J Trop Med Hyg. 1981;30:1291–3. 10.4269/ajtmh.1981.30.1291 7034562

[11] Lecompte E, ter Meulen J, Emonet S, Daffis S, Charrel RN. Genetic identification of Kodoko virus, a novel arenavirus of the African pigmy mouse (Mus Nannomys minutoides) in West Africa. Virology. 2007;364:178–83. 10.1016/j.virol.2007.02.008 17382366

[12] de Bellocq JG, Borremans B, Katakweba A, Makundi R, Baird SJ, Becker-Ziaja B, et al. Sympatric occurrence of 3 arenaviruses, Tanzania. Emerg Infect Dis. 2010;16:692–5. 10.3201/eid1604.091721 20350390 PMC3321973

[13] Bowen MD, Peters CJ, Nichol ST. The phylogeny of New World (Tacaribe complex) arenaviruses. Virology. 1996;219:285–90. 10.1006/viro.1996.0248 8623541

[14] Kumar S, Stecher G, Tamura K. MEGA7: Molecular Evolutionary Genetics Analysis version 7.0 for bigger datasets. Mol Biol Evol. 2016;33:1870–4. 10.1093/molbev/msw054 27004904 PMC8210823

[15] Chimimba CT, Bennett NC. 2005. Order Rodentia. In: Skinner JD and Chimimba CT, editors. The mammals of the southern African subregion, 3rd edition. Cape Town: Cambridge University Press; 2005. p. 77–209.

[16] Russo IR, Chimimba CT, Bloomer P. Bioregion heterogeneity correlates with extensive mitochondrial DNA diversity in the Namaqua rock mouse, *Micaelamys namaquensis* (Rodentia: Muridae) from southern Africa—evidence for a species complex. BMC Evol Biol. 2010;10:307. 10.1186/1471-2148-10-307 20942924 PMC2967545

[17] Do Linh San E, Babu N, Xalu M, Le Gars S, Perquin J-C, Baxter RM, et al. A conservation assessment of Otomys unisulcatus. In: Child MF, Roxburgh L, Do Linh San E, Raimondo D, Davies-Mostert HT, editors. The red list of mammals of South Africa, Swaziland and Lesotho 2016. Pretoria, South Africa: South African National Biodiversity Institute and Endangered Wildlife Trust; 2017.

[18] Monadjem A, Taylor PJ, Denys C, Cotterill FPD. Rodents of sub-Saharan Africa: a biogeographic and taxonomic synthesis. Berlin: Walter de Gruyter GmbH; 2015.

[19] Castiglia R, Solano E, Makundi RH, Hulselmans J, Verheyen E, Colangelo P. Rapid chromosomal evolution in the mesic four-striped grass rat *Rhabdomys dilectus* (Rodentia, Muridae) revealed by mtDNA phylogeographic analysis. J Zool Syst Evol Res. 2011;50:165–72. 10.1111/j.1439-0469.2011.00627.x

[20] du Toit N, van Vuuren BJ, Matthee S, Matthee CA. Biome specificity of distinct genetic lineages within the four-striped mouse *Rhabdomys pumilio* (Rodentia: Muridae) from southern Africa with implications for taxonomy. Mol Phylogenet Evol. 2012;65:75–86. 10.1016/j.ympev.2012.05.036 22728170

[21] Ganem G, Dufour C, Avenant N, Caminade P, Eiseb S, Tougard C, et al. An update on the distribution and diversification of *Rhabdomys sp*. (Muridae, Rodentia). J Vert Biol. 2020;69:1. 10.25225/jvb.20013

[22] McIntosh BM, Dickinson DB, Meenehan GM, Dos Santos IS. *Culex (Eumelanomyia) rubinotus* Theobald as vector of Banzi, Germiston and Witwatersrand viruses. II. Infections in sentinel hamsters and wild rodents. J Med Entomol. 1976;12:641–4. 10.1093/jmedent/12.6.641 1263212

[23] Monath TP, Newhouse VF, Kemp GE, Setzer HW, Cacciapuoti A. Lassa virus isolation from *Mastomys natalensi*s rodents during an epidemic in Sierra Leone. Science. 1974;185:263–5. 10.1126/science.185.4147.263 4833828

[24] Walker DH, Johnson KM, Lange JV, Gardner JJ, Kiley MP, McCormick JB. Experimental infection of rhesus monkeys with Lassa virus and a closely related arenavirus, Mozambique virus. J Infect Dis. 1982;146:360–8. 10.1093/infdis/146.3.360 6286795

[25] Paweska JT, Sewlall NH, Ksiazek TG, Blumberg LH, Hale MJ, Lipkin WI, et al.; Outbreak Control and Investigation Teams. Nosocomial outbreak of novel arenavirus infection, southern Africa. Emerg Infect Dis. 2009;15:1598–602. 10.3201/eid1510.090211 19861052 PMC2866397

[26] Palacios G, Savji N, Hui J, Travassos da Rosa A, Popov V, Briese T, et al. Genomic and phylogenetic characterization of Merino Walk virus, a novel arenavirus isolated in South Africa. J Gen Virol. 2010;91:1315–24. 10.1099/vir.0.017798-0 20071489 PMC2888150

[27] Colangelo P, Verheyen E, Leirs H, Tatard C, Denys C, Dobigny G, et al. A mitochondrial phylogeographic scenario for the most widespread African rodent, Mastomys natalensis. Biol J Linn Soc Lond. 2013;108:901–16. 10.1111/bij.12013

[28] Göuy de Bellocq J, Bryjová A, Martynov A, Lavrenchenko L. Dhati Welel virus, the missing mammarenavirus of the widespread Mastomys natalensis. J Vert Biol. 2020;69:20018. 10.25225/jvb.20018

[29] Gryseels S, Baird SJE, Borremans B, Makundi R, Leirs H, Goüy de Bellocq J. When viruses don’t go viral: the importance of host phylogeographic structure in the spatial spread of arenaviruses. PLoS Pathog. 2017;13:e1006073. 10.1371/journal.ppat.1006073 28076397 PMC5226678

[30] Radoshitzky SR, Buchmeier MJ, Charrel RN, Clegg JCS, Gonzalez JJ, Günther S, et al.; Ictv Report Consortium. ICTV virus taxonomy profile: Arenaviridae. J Gen Virol. 2019;100:1200–1. 10.1099/jgv.0.001280 31192784 PMC12139605

[31] Witkowski PT, Kallies R, Hoveka J, Auste B, Ithete NL, Šoltys K, et al. Novel arenavirus isolates from Namaqua rock mice, Namibia, Southern Africa. Emerg Infect Dis. 2015;21:1213–6. 10.3201/eid2107.141341 26079174 PMC4480381

[32] Těšíková J, Krásová J, Goüy de Bellocq J. Multiple mammarenaviruses circulating in Angolan rodents. Viruses. 2021;13:982. 10.3390/v13060982 34070551 PMC8227972

[33] Edwards S, Claude J, Van Vuuren BJ, Matthee CA. Van Vuuren Bj, Matthee Ca. Evolutionary history of the Karoo bush rat, Myotomys unisulcatus (Rodentia: Muridae): disconcordance between morphology and genetics. Biol J Linn Soc Lond. 2011;102:510–26. 10.1111/j.1095-8312.2010.01583.x

[34] Ishii A, Thomas Y, Moonga L, Nakamura I, Ohnuma A, Hang’ombe BM, et al. Molecular surveillance and phylogenetic analysis of Old World arenaviruses in Zambia. J Gen Virol. 2012;93:2247–51. 10.1099/vir.0.044099-0 22815269

